# *SHMT1* C1420T polymorphism contributes to the risk of non-Hodgkin lymphoma: evidence from 7309 patients

**DOI:** 10.1186/s40880-015-0065-z

**Published:** 2015-12-14

**Authors:** Yi-Wei Wang, Shao-Dan Zhang, Wen-Ji Xue, Mei-Ling Zhu, Lei-Zhen Zheng

**Affiliations:** Department of Oncology, Xin Hua Hospital Affiliated To Shanghai Jiaotong University School of Medicine, NO. 1665, Kong Jiang Road, Shanghai, 200092 P.R. China; Department of Experimental Research, State Key Laboratory of Oncology in South China; Collaborative Innovation Center for Cancer Medicine, Sun Yat-sen University Cancer Center, Guangzhou, 510060 Guangdong P.R. China

**Keywords:** Serine hydroxymethyltransferase 1 (SHMT1), Polymorphism, Non-Hodgkin lymphoma, Meta-analysis

## Abstract

**Background:**

Serine hydroxymethyltransferase 1 (SHMT1) is a key enzyme in the folate metabolic pathway that plays an important role in biosynthesis by providing one carbon unit. *SHMT1* C1420T may lead to the abnormal biosynthesis involved in DNA synthesis and methylation, and it may eventually increase cancer susceptibility. Many epidemiologic studies have explored the association between C1420T polymorphism and the risk of non-Hodgkin lymphoma (NHL), but the results have been contradictory. Therefore, we performed this meta-analysis to evaluate the relationship.

**Methods:**

The meta-analyses were conducted to evaluate the effect of *SHMT1* C1420T polymorphism on NHL risk. Odds ratios (ORs) and 95% confidence intervals (CIs) were calculated to measure the strength of the association.

**Results:**

Eight studies encompassing 3232 cases and 4077 controls were included. A statistically significant association was found between *SHMT1* C1420T polymorphism and NHL risk under the allelic comparison (T vs. C: OR = 1.09, 95% CI 1.01–1.17); a borderline association was found between *SHMT1* C1420T polymorphism and NHL risk under the homozygote model (TT vs. CC: OR = 1.18, 95% CI 1.00–1.39) and the dominant model (CT+TT vs. CC: OR = 1.10, 95% CI 1.00–1.21).

**Conclusion:**

*SHMT1* C1420T polymorphism may be associated with NHL risk, which needs to be validated in large, prospective studies.

## Background

In the past 30 years, the incidence of non-Hodgkin lymphoma (NHL), a common hematologic malignancy, has increased markedly [1, 2]. Generally, there are two major types of NHL: B cell lymphomas and T-cell lymphomas, with B-cell lymphomas accounting for the majority (approximately 85%) of cases. Diffuse large B-cell lymphoma (DLBCL) and follicular lymphoma (FL) are two major subtypes of B-cell lymphomas [3, 4]. Risk factors for NHL include family history, immune dysfunction, immune stimulation, and environmental exposures such as infection, high doses of radiation and pesticides [5, 6]. In addition, although the underlying biological mechanisms involved in NHL remain unidentified, it has been shown that chromosomal and genetic alterations, caused by the total influence of multiple single nucleotide polymorphisms (SNPs) in the genes implicated in various molecular pathways, also play an important role in the development of NHL [7–9]. For example, folate-metabolizing genes play significant roles in the development of NHL [8]. Therefore, genetic variability in folate-metabolizing genes may be closely related to NHL risk.

Serine hydroxymethyltransferase (SHMT), a key enzyme involved in the folate metabolism, can reversibly catalyze serine and tetrahydrofolate to glycine and 5,10-methylenetetrahydrofolate [10]. SHMT has two distinct isoenzymes, one locating in the cytoplasm (SHMT1) and the other locating in mitochondria (SHMT2). *SHMT1,* localized on chromosome 17p11.2 [11], plays a key role in inducing gene methylation and DNA synthesis by providing one-carbon atoms for purine, thymidylate, and methionine in the cytoplasm [12]. Abnormal methylation and DNA repair systems may cause genome instability and lead to overexpression of oncogenes and inactivation of tumor suppressor genes [13, 14], which closely relate to the occurrence and development of common tumors [15]. Consequently, abnormally functioning *SHMT1* can affect cell progression and ultimately cause cancer. One SNP has been found at nucleotide 1420 (C1420T, rs1979277) [16], and it can influence the function of *SHMT1* by a leucine-to-phenylalanine amino acid substitution at codon 474 (Leu474Phe) of the protein [17]. Hence, in people who carry the mutation, the NHL risk might be higher than those without the mutation.

To date, many studies have investigated the association between *SHMT1* C1420T polymorphism and NHL risk, but the conclusions are mixed rather than conclusive, partially because of possible weak effects of the polymorphisms on NHL risk, the relatively small sample size in each previous investigation, or the patients’ diverse racial backgrounds. Therefore, we developed a comprehensive meta-analysis of all eligible case–control studies to derive a more precise risk estimate for the association.

## Methods

### Literature search strategy

We searched two electronic databases (PubMed and Embase) to identify all published studies with the following terms: “*SHMT*”, “*SHMT1*”, or “cytosolic serine hydroxymethyltransferase”, “polymorphism” or “variant”, “non-Hodgkin lymphoma” or “NHL”, and “cancer”, “neoplasia”, or “malignancy” (last search date: August 1, 2015). To expand the scope of our search, we also searched the Chinese National Knowledge Infrastructure database (CNKI) with the terms “*SHMT*”, “*SHMT1*”, and ‘‘NHL’’ in Chinese. Furthermore, we manually searched reference lists on this topic to identify additional relevant studies and attempted to contact the authors for more information if the information available was incomplete.

### Selection criteria

The studies selected in this meta-analysis had to meet the following criteria: (1) be written in English or Chinese; (2) have a case–control design; (3) evaluate the association between *SHMT1* C1420T polymorphism and NHL risk; and (4) provide sufficient data for the calculation of odds ratios (ORs) and 95% confidence intervals (CIs). Abstracts and unpublished reports were excluded. Moreover, if studies had the same subjects or overlapping data, we selected the one with the largest sample size.

### Data extraction

Two reviewers independently abstracted the following information from each study according to standardized criteria: first author, year of publication, country of population studied, ethnicity of population studied, NHL subtype (DLBCL or FL), source of controls (population-based, hospital-based, or mixed), total number of genotyped cases and controls, and numbers of genotypes (CC, CT, and TT) for the C1420T polymorphism in cases and controls. If any different views existing, we discussed it until consensus was reached.

### Statistical methods

We used the crude ORs and 95% CIs to determine the association between *SHMT1* C1420T polymorphism and NHL risk under different genetic models as follows: allelic comparison (T vs. C), homozygote model (TT vs. CC), heterozygote model (CT vs. CC), recessive model (TT vs. CC+CT), and dominant model (CT+TT vs. CC). Additionally, we performed stratification analyses by tumor subtype (DLBCL and FL) and by patient ethnicity (Caucasian and mixed; if the genotyping data listed in one article were for a mixture of different populations, this article was marked as “mixed” ethnicity). Goodness-of-fit Chi-square test was used to evaluate deviation from the Hardy–Weinberg equilibrium (HWE) for the genotypes of controls. The Chi-square-based *Q* test was performed to calculate inter-study heterogeneity. If *P* < 0.05, we used the random-effects model to assess the pooled ORs because this model tends to provide wider 95% CIs [18]; otherwise, we used the fixed-effects model [19]. Furthermore, we conducted sensitivity analyses to evaluate the influence of individual studies on NHL risk by excluding one study sequentially each time and recalculating the pooled ORs and their 95% CIs. Moreover, publication bias was examined by the inverted funnel plot and the Egger test, and an asymmetric plot or *P* < 0.05 as determined by the Egger test was considered statistically significant [20]. All analyses were performed using the Review Manager software version 5.2.22.0 (The Nordic Cochrane Centre, The Cochrane Collaboration, London, UK). All tests were two-sided, and *P* < 0.05 was considered statistically significant.

## Results

### Study characteristics

**Fig. 1:**
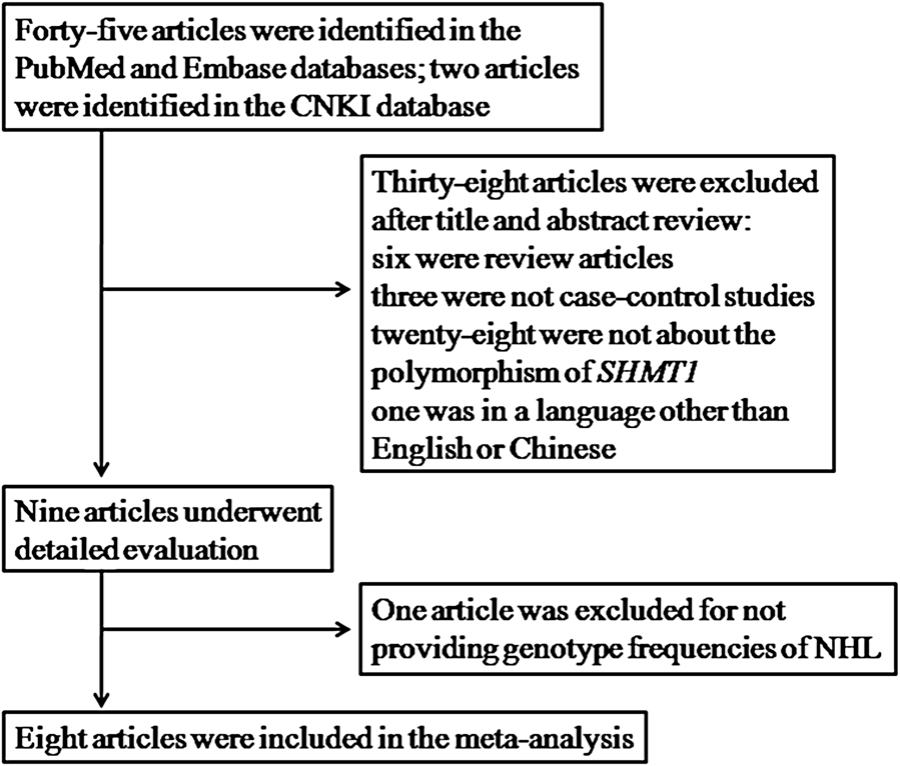
Flow diagram of studies included in this meta-analysis. *CNKI* China National Knowledge Infrastructure, *SMHT1* serine hydroxymethyltransferase 1 and *NHL* non-Hodgkin lymphoma

After initial screening, we found 47 relevant publications. We excluded 38 of these studies after reviewing the titles and abstracts (6 were review articles, 3 were not case–control studies, 28 were not about the *SHMT1* polymorphism, and 1 was in a language other than English or Chinese), and 9 articles were left for full review. Of these, 1 study was excluded for not providing genotype frequencies of NHL [21]. Eventually, 8 studies met our inclusion criteria; these studies covered 3232 NHL cases and 4077 controls, all of which were included in our pooled analyses [22–29] (Fig. 1). Table 1 lists the essential characteristics for all included studies. Of the 8 studies included, only 5 examined the association between the *SHMT1* C1420T polymorphism and the subtype of NHL risk [22, 23, 25, 26, 29]. Of these 5 studies, 4 studies [22, 23, 25, 26] with 744 cases included 2353 controls for the DLBCL subtype, and 5 studies [22, 23, 25, 26, 29] with 778 cases included 2558 controls for the FL subtype. Additionally, 6 studies were conducted in Caucasian patients; the remaining 2 studies were conducted in patients from mixed ethnic groups. All studies were population-based designed; 7 studies of genotype distribution in the controls were in line with HWE except the study conducted by Li et al. [28].

**Table 1:** Characteristics of the 8 publications included in the meta-analysis

Study^a^	Country	Ethnicity	Source of controls	Sample sizes (cases/controls)	HWE (controls)	MAF
Skibola (2004) [22]	USA	Caucasian	Population-based	333/729	0.509	0.32
Lightfoot (2005) [23]	USA	Caucasian	Population-based	589/754	0.181	0.32
Lee (2007) [24]	Australia	Caucasian	Population-based	553/498	0.129	0.32
Lim (2007) [25]	USA	Mixed^b^	Population-based	743/629	0.283	0.31
Berglund (2009) [26]	Sweden	Caucasian	Population-based	258/241	0.630	0.32
Weiner (2011) [27]	Russia	Caucasian	Population-based	141/504	0.357	0.33
Li (2013) [28]	USA	Mixed^b^	Population-based	446/517	0.044	0.30
Niclot (2006) [29]	France	Caucasian	Population-based	169/205	0.547	0.31

*USA* the United States of America, *HWE* Hardy–Weinberg equilibrium, *MAF* minor allele frequency

^a^Each study is presented as the first author’s last name followed by the year of publication

^b^Mixed: in the study, the genotyping data were mixed from different ethnic populations

### Quantitative synthesis

**Fig. 2:**
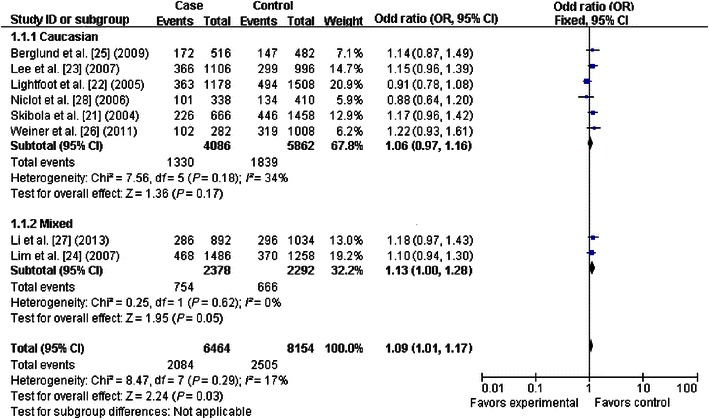
Forest plot for NHL risk associated with the *SHMT1* C1420T polymorphism under the0020allelic comparison (T vs. C), stratified by ethnicity. A significant association was detected between the *SHMT1* C1420T polymorphism and NHL risk under the allelic comparison. The *boxes* and *horizontal lines* correspond to the estimates of odds ratio (OR) and 95% confidence interval (CI) for each study. The *diamond* indicates the pooled OR and 95% CI

**Fig. 3:**
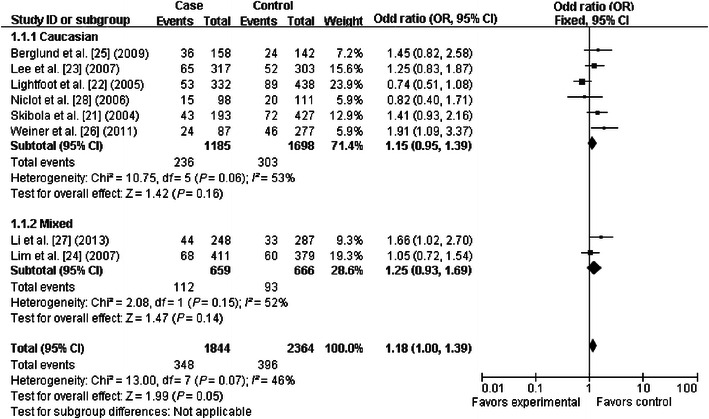
Forest plot for NHL risk associated with the *SHMT1* C1420T polymorphism under the homozygote model (TT vs. CC), stratified by ethnicity. A borderline association was detected between the *SHMT1* C1420T polymorphism and NHL risk under the homozygote model. The *boxes* and *horizontal lines* correspond to the estimates of OR and 95% CI for each study. The *diamond* indicates the pooled OR and 95% CI

**Fig. 4:**
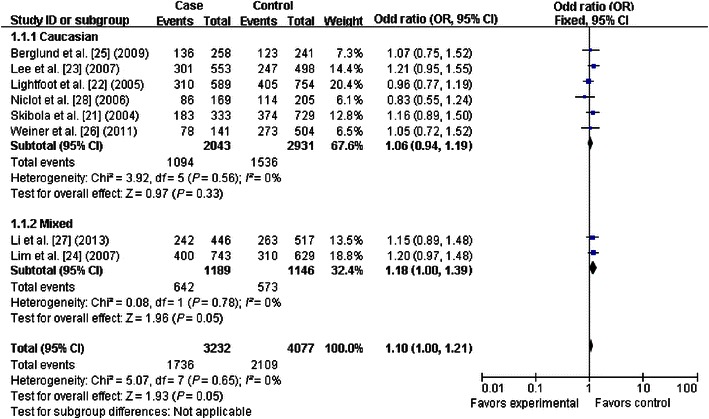
Forest plot for NHL risk associated with the *SHMT1* C1420T polymorphism under the dominant model (CT+TT vs. CC), stratified by ethnicity. A borderline association was detected between the *SHMT1* C1420T polymorphism and NHL risk under the dominant model. The *boxes* and *horizontal lines* correspond to the estimates of OR and 95% CI for each study. The *diamond* indicates the pooled OR and 95% CI

**Fig. 5:**
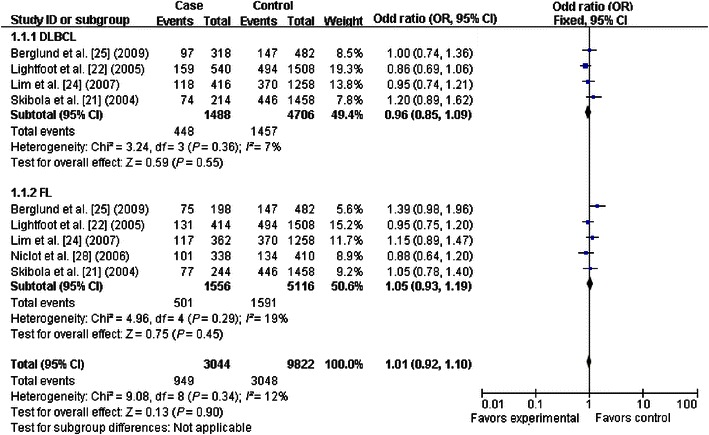
Forest plots for NHL risk associated with the *SHMT1* C1420T polymorphism, stratified by NHL type (T vs. C). No significant association was detected in the stratification analysis by NHL subtype. The *boxes* and *horizontal lines* correspond to the estimates of OR and 95% CI for each study. The *diamond* indicates the pooled OR and 95% CI

**Fig. 6:**
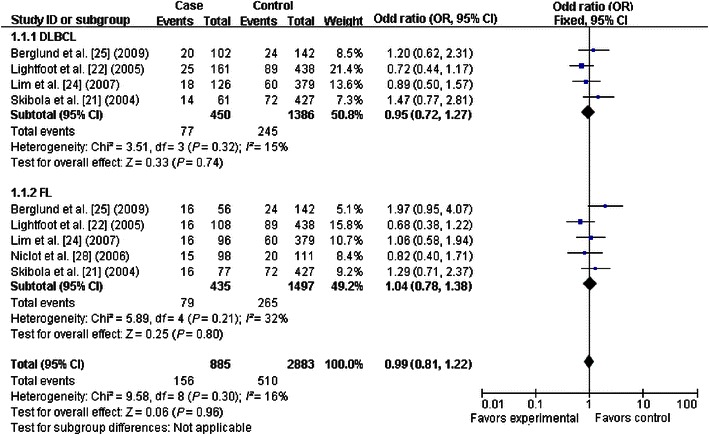
Forest plots for NHL risk associated with the *SHMT1* C1420T polymorphism, stratified by NHL type (TT vs. CC). No significant association was detected in the stratification analysis by NHL subtype. The *boxes* and *horizontal lines* correspond to the estimates of OR and 95% CI for each study. The *diamond* indicates the pooled OR and 95% CI

**Fig. 7:**
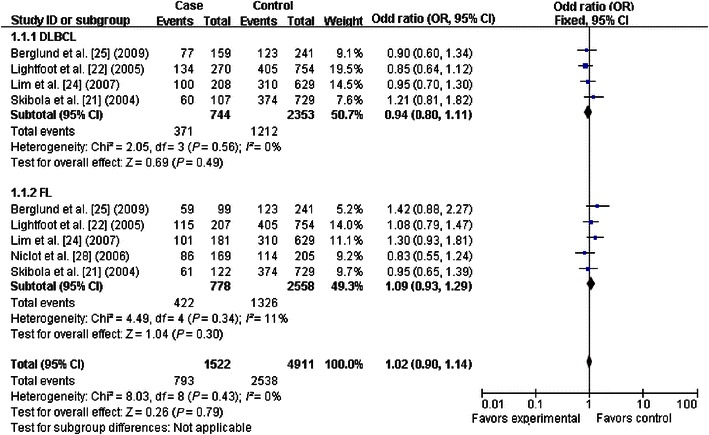
Forest plots for NHL risk associated with the *SHMT1* C1420T polymorphism, stratified by NHL type (CT+TT vs. CC). No significant association was detected in the stratification analysis by NHL subtype. The *boxes* and *horizontal lines* correspond to the estimates of OR and 95% CI for each study. The *diamond* indicates the pooled OR and 95% CI. *DLBCL* diffuse large B-cell lymphoma and *FL* follicular lymphoma

The primary results of this meta-analysis are presented in Table 2 and Figs. 2, 3, 4, 5, 6 and 7. In the pooled analysis, we found a statistically significant association between *SHMT1* C1420T polymorphism and NHL risk under the allelic comparison (T vs. C: OR = 1.09, 95% CI 1.01–1.17); we found a borderline association between *SHMT1* C1420T polymorphism and NHL risk under the homozygote model (TT vs. CC: OR = 1.18; 95% CI 1.00–1.39) and under the dominant model (CT+TT vs. CC: OR = 1.10; 95% CI 1.00–1.21). In the subgroup analysis by ethnicity, we found no association for Caucasians but a borderline direct association for mixed ethnic subgroups under the allelic comparison (T vs. C: OR = 1.13; 95% CI 1.00–1.28) and the dominant model (CT+TT vs. CC: OR = 1.18; 95% CI 1.00–1.39).

**Table 2:** Meta-analysis of the association between *SHMT1* C1420T polymorphism and non-Hodgkin lymphoma (NHL) risk

Variable	Number of studies	Cases/controls	T vs. C			TT vs. CC			CT+TT vs. CC			CT vs. CC			TT vs. CC+CT		
			OR (95% CI)^a^	*P*	*P*_het_	OR (95% CI)^a^	*P*	*P*_het_	OR (95% CI)^a^	*P*	*P*_het_	OR (95% CI)^a^	*P*	*P*_het_	OR (95% CI)^b^	*P*	*P*_het_
All	8	3232/4077	*1.09* (*1.01*–*1.17*)	0.025	0.293	*1.18* (*1.00*–*1.39*)	0.046	0.072	*1.10* (*1.00*–*1.21*)	0.054	0.652	1.08 (0.98–1.19)	0.140	0.640	1.19 (0.94–1.50)	0.144	0.035
Ethnicity																	
Caucasian	6	2043/2931	1.06 (0.97–1.16)	0.174	0.182	1.15 (0.95–1.39)	0.156	0.057	1.06 (0.94–1.19)	0.332	0.561	1.04 (0.92–1.17)	0.574	0.647	1.19 (0.88–1.60)	0.252	0.033
Mixed	2	1189/1146	*1.13* (*1.00*–*1.28*)	0.051	0.620	1.25 (0.93–1.69)	0.141	0.149	*1.18* (*1.00*–*1.39*)	0.050	0.784	1.16 (0.98–1.38)	0.081	0.423	1.21 (0.73–2.01)	0.458	0.087
Subtype																	
DLBCL	4	744/2353	0.96 (0.85–1.09)	0.555	0.357	0.95 (0.72–1.27)	0.744	0.319	0.94 (0.80–1.11)	0.490	0.562	0.94 (0.79–1.12)	0.490	0.717	0.98 (0.75–1.29)	0.895	0.370
FL	5	778/2558	1.05 (0.93–1.19)	0.454	0.292	1.04 (0.78–1.38)	0.804	0.208	1.09 (0.93–1.29)	0.343	0.298	1.10 (0.93–1.31)	0.263	0.297	0.99 (0.75–1.29)	0.926	0.156
Publication bias^c^			0.829			0.389			0.290			0.024			0.234		

The results were in italics, if the 95% CI excluded 1 or *P* < 0.05

*P*_het_ value of the *Q* test for heterogeneity

*DLBCL* diffuse large B-cell lymphoma, *FL* follicular lymphoma, *OR,* odds ratio, *CI* confidence interval

^a^Fixed-effects model

^b^Random-effects model

^c^ *P* value of the Egger test for publication bias

After excluding the study by Li et al. [28] because the genotype frequencies in the controls deviated from HWE, we conducted further analysis and determined that the positive result was converted into a negative one. No significant association was found in the stratification analysis by tumor subtype.

We calculated statistical power to detect an OR of 1.50 for a risk effect, with a level equal to the observed *P* value. We further used the false-positive report probability (FPRP) with prior probabilities of 0.0001, 0.001, 0.01, 0.1, and 0.25 to account for chance associations from multiple comparisons (Table 3). Results with an FPRP value less than 0.20 were considered significant associations [30].

**Table 3:** False-positive report probability values and statistical power for associations between genotypes of *SHMT1* C1420T polymorphism and NHL risk

Genotype	Positive OR (95% CI)^a^	*P* value^b^	Statistical power^c^	Prior probability				
				0.25	0.10	0.01	0.001	0.0001
*SHMT1* C1420T								
T vs. C	1.09 (1.01–1.17)	0.025	1.000	0.070	0.184	0.712	0.962	0.996
TT vs. CC	1.18 (1.00–1.39)	0.046	0.999	0.121	0.293	0.820	0.979	0.998
CT+TT vs. CC	1.10 (1.00–1.21)	0.054	0.820	0.165	0.372	0.867	0.985	0.999

Abbreviations as in Tables 1 and 2

^a^The OR reported in Table 2

^b^Genotype frequency distributions were calculated using the omnibus Chi-square test in Table 2

^c^Statistical power was calculated using the number of observations (cases and controls) and the OR and *P* values in Table 2

### Heterogeneity and sensitivity analyses

No significant between-study heterogeneities were observed among the overall studies for the association of *SHMT1* C1420T polymorphism with NHL risk (allelic comparison: *P* = 0.29; homozygote model: *P* = 0.07; dominant model: *P* = 0.65; heterozygote model: *P* = 0.64), except for the recessive model (*P* = 0.04). In the sensitivity analyses, the results indicated that a single study might change the pooled ORs (data not shown).

### Publication bias

Begg’s funnel plot and the Egger test were used to evaluate the publication bias of all included studies. The shapes of the funnel plots appeared to be symmetrical, and the Egger test further suggested that there was no significant evidence of publication bias under some genetic models (allele comparison: *P* = 0.83; homozygote model: *P* = 0.39; dominant model: *P* = 0.29; recessive model: *P* = 0.23), but the heterozygote model showed significant publication bias (*P* = 0.02).

## Discussion

Our meta-analysis, which examined eight studies encompassing 3232 NHL cases and 4077 controls, investigated the association between *SHMT1* C1420T polymorphism and NHL risk. A borderline association was detected, which indicated that this polymorphism may increase NHL risk, although the effect of the SNP was very weak.

SHMT1 is a key enzyme in the folate metabolic pathway and supplies one-carbon molecules to the cycle; this carbon plays an important role in the biosynthesis of purine, thymidylate, and methionine [12], which are essential for DNA synthesis and gene methylation. Therefore, the 1420 C>T polymorphism in *SHMT1* creates an imbalance in folate metabolism, which adversely affects DNA synthesis and methylation systems and causes genome instability, eventually leading to overexpression of oncogenes and inactivation of tumor suppressor genes [13, 14]. Additionally, the polymorphism can cause reduced circulating folate levels [23], which not only shunts 5,10-methylenetetrahydrofolate toward DNA synthesis but also results in uracil misincorporation into DNA, eventually leading to double-strand breaks, chromosomal damage, and cancer [31, 32].

We found that *SHMT1* C1420T might have a weak effect on NHL risk. There are several possible explanations for this result. First, because only eight studies met our review criteria, the sample size of the meta-analysis was not large enough to detect a specific effect on NHL risk. Second, the cancer risk conferred by the genetic variation is indeed very modest, and the penetrance is very small for the variants. Third, other causal genes, which are implicated in the pathogenesis of cancer, might mask the effect of *SHMT1* C1420T polymorphisms by gene–gene interactions and, consequently, modulate cancer susceptibility. In any case, studies with larger sample sizes are warranted to validate our findings.

In the stratifying analysis by ethnicity, we found that *SHMT1* C1420T polymorphism might be associated with an increased NHL risk in the mixed ethnic group but not in the Caucasian group, suggesting that there are some differences in genetic information of individuals from different races. In addition, the possibility of misinformation was not ruled out. However, because of the small sample size of the mixed ethnic subgroup, this result remains questionable, and additional studies with larger sample sizes are needed. To date, only one meta-analysis, which was published in 2011 [26], has focused on the association between *SHMT1* C1420T and NHL risk. After examining eight studies that encompassed 2884 cases and 4054 controls, Weiner et al. [27] concluded that *SHMT1* C1420T had no effect on the risk of NHL, which was inconsistent with the results of our study. Have been examined carefully, we found that the meta-analysis by Weiner et al. [27] included one study by Hishida et al. [33] that we excluded because it provided the data about malignant lymphoma rather than NHL. In addition, we added one more study [29] in our meta-analysis to enlarge the sample size and improve the statistical power. Therefore, compared with the meta-analysis by Weiner et al. [27], our study derives a more precise risk estimate for the association between *SHMT1* C1420T polymorphism and NHL risk. Furthermore, we put all the studies together to collectively analyze and then draw a conclusion. Although researchers commonly pool data from different cancer subtypes, it is unclear whether the association of genetic variation with risk of cancer should be calculated this way. We used this approach because many studies have discovered that some sequence variants in specific regions of chromosomes, such as 17p11.2, are associated with risk of specific subtypes of cancer [28, 34]. We speculate that the SNP may be the specific site associated with different NHL subtypes.

Our meta-analysis has several limitations. First, the results of these sensitivity analyses indicated that a single study might change the pooled ORs, which means that our results may have low robustness and should be interpreted cautiously. Second, selection bias resulted from the fact that only studies written in English or Chinese were included in this meta-analysis. Third, the sample size of the included studies was relatively small, especially in the stratified analysis, which may result in limited statistical power. Fourth, significant heterogeneity in the meta-analysis was observed under the recessive model. We did not deem lightly the issue of the random-effects model used to incorporate heterogeneity among studies. Further stratification analysis suggested that ethnicity may be the main source of heterogeneity. Moreover, there is other heterogeneity that cannot be explained. Fifth, because more detailed information was not available in the included studies, possible compounding factors (such as age and sex) could not be obtained for stratification analysis to further evaluate the relationship between *SHMT1* polymorphism and NHL risk. Sixth, because all included studies were case–control, this may have caused selection bias, implementation bias, and confounding bias because of the nature of retrospective studies. Finally, in terms of publication bias, the funnel plot’s power is relatively low when fewer than 30 publications are tested for asymmetry. Moreover, most of the data on publication bias are retrospective rather than prospective, including our current analysis. Reporting publication bias from prospective studies is necessary.

In summary, we found in this updated meta-analysis that *SHMT1* C1420T polymorphism may be a risk factor for NHL. Additional well-designed studies with larger sample sizes and more information about confounding factors are needed to validate our findings.
